# An Insight into the “-Omics” Based Engineering of Streptomycetes for Secondary Metabolite Overproduction

**DOI:** 10.1155/2013/968518

**Published:** 2013-09-02

**Authors:** Amit Kumar Chaudhary, Dipesh Dhakal, Jae Kyung Sohng

**Affiliations:** Department of Pharmaceutical Engineering, Institute of Biomolecule Reconstruction, SunMoon University, 100 Kalsan-ri, Tangjeongmyeon, Asan-si, Chungnam 336-708, Republic of Korea

## Abstract

Microorganisms produce a range of chemical substances representing a vast diversity of fascinating molecular architectures not available in any other system. Among them, *Streptomyces* are frequently used to produce useful enzymes and a wide variety of secondary metabolites with potential biological activities. *Streptomyces* are preferred over other microorganisms for producing more than half of the clinically useful naturally originating pharmaceuticals. However, these compounds are usually produced in very low amounts (or not at all) under typical laboratory conditions. Despite the superiority of *Streptomyces*, they still lack well documented genetic information and a large number of in-depth molecular biological tools for strain improvement. Previous attempts to produce high yielding strains required selection of the genetic material through classical mutagenesis for commercial production of secondary metabolites, optimizing culture conditions, and random selection. However, a profound effect on the strategy for strain development has occurred with the recent advancement of whole-genome sequencing, systems biology, and genetic engineering. In this review, we demonstrate a few of the major issues related to the potential of “-omics” technology (genomics, transcriptomics, proteomics, and metabolomics) for improving streptomycetes as an intelligent chemical factory for enhancing the production of useful bioactive compounds.

## 1. Introduction

Natural products are chemical compounds with pharmacological characteristics produced by living organisms that can be utilized during pharmaceutical drug discovery, agriculture, and in the food industry. They are called “secondary metabolites,” as they can be synthesized by microorganisms and plants but are not essential for their own metabolic processes [1]. Microbial fermentation is widely applied to industrially produce these valuable compounds. Usually, these compounds are produced in very low amounts (or not at all) by natural strains under typical laboratory conditions to meet commercial requirements, demonstrating the need for heterologous expression of these biosynthetic gene clusters. Of the thousands of secondary metabolites documented, more than half are produced by *Streptomyces *(antibiotics, antitumor agents, immunosuppressants, antihelminthics, antifungals, herbicides, and insecticides) and have offered decades of interest to industry and academia [2, 3] (Table 1). Besides, many of these secondary metabolites function as signaling molecules to control the metabolism of their own producer [4]. This vast reservoir of diverse products makes streptomycetes the most important industrial microbial genus. Based on the Waksman and Henrici classification of 1943, these organisms are classified in the family Streptomycetaceae[5]. They are Gram-positive mycelial soil bacteria containing about 70% G-C DNA content and undergo a complex process of morphological development that usually involves secondary metabolite biosynthesis under depleted nutrient conditions [6]. 

Genome mining of several *Streptomyces* [7–10] has revealed numerous cryptic novel secondary metabolite biosynthetic gene clusters, which encode the potential to synthesize a large diversity of compounds that have never been observed before, including polyketides, aminoglycosides, bacteriocins, terpenoids, shikimate-derived metabolites, nonribosomal peptides, anthracyclines, macrolides, beta-lactams, and other natural products [11]. The core unit responsible for secondary metabolite production is specifically termed a biosynthetic gene cluster, which encompasses biosynthetic enzymes, resistance determinants, and regulatory proteins. The use of these cryptic microbial secondary metabolic processes has attracted the attention of synthetic microbiologists who exploit recent advances in DNA sequencing and synthesis procedures to achieve unprecedented control over metabolic pathways. In particular, rare *Streptomyces* are promising sources for new drugs; therefore, genetic manipulations of these microorganisms are crucial for drug discovery and development. 

**Figure 1:**
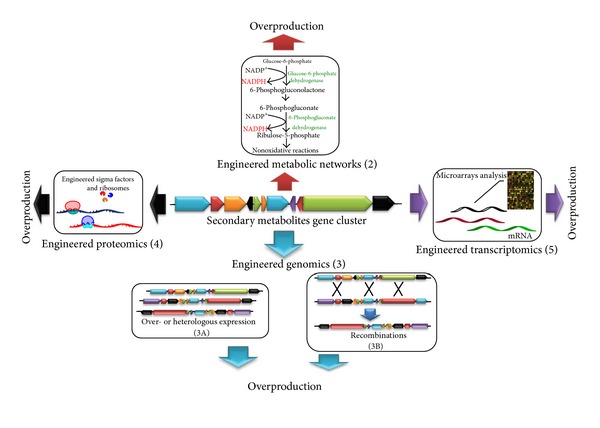
The approaches used for overproduction of secondary metabolite in Streptomycetes.

Recent advances in “-omics” technologies introduced during the last two decades have allowed the establishment of various research areas pertaining to *Streptomyces*. In general, “-omics” considers versatile genes and their products such as transcripts, proteins, or metabolites. Genomics [12] deals with genes, their variation, and function, whereas transcriptomics considers information at the mRNA (transcript) level at a particular time depending on environmental signals and biophysiological parameters. Proteomics considers expression, function, and regulation of an entire set of proteins [13]. Moreover, as the proteins within cells are the functional units, their expression is strongly influenced by environmental signals and physiological conditions, and, thus, proteomics is a complementary technology to genomic and transcriptomic research [14]. Metabolomics encompasses detailed metabolic analysis [15]. Since the genome of the first cellular organism *Haemophilus influenza *was sequenced [16], the availability of metabolic network models has helped develop several computational approaches for flux balance analyses [17]. However, relatively little is known about the metabolic pathways of most *Streptomyces*, but an extensive review on primary metabolism reported that the Embden-Meyerhof-Parnas, the pentose phosphate (PPP), and tricarboxylic acid cycle pathways are present in a number of *Streptomyces* species. [18]. Based on the huge progress in “-omics” technologies, new approaches are aimed at ensuring optimal engineering of the cell factory to achieve optimized metabolite production [19] (Figure 1). In this review, we illustrate the pivotal roles of genomics, transcriptomics, proteomics, and metabolomics as research tools in systems biology of secondary metabolism for enhancing the production of secondary metabolites in streptomycetes. We discuss the potential of exploiting “-omics” tools to enhance the production of naturally originating pharmaceuticals by circumventing major bottlenecks to overproduce compounds of interest (Tables 2, 3, 4 and 5). Furthermore, we conclude the paper by highlighting the future perspectives of recent technological advances yet to be applied to *Streptomyces* species for further stain improvement.

## 2. Engineered Metabolic Networks

Among the “-omics” technologies [12–15], one important tool in the system biology toolbox is metabolomics, which catalogues all small metabolites in a biological sample [20, 21]. It is expected to play a significant role in bridging the phenotype-genotype gap, as it amplifies changes in the proteome and provides a better representation of an organism's phenotype. Moreover, knowledge of the complete set of metabolites provides the intracellular fluxes required for a comprehensive characterization of metabolic networks and their operation. Therefore, intracellular fluxes can help in high level production of pharmaceuticals requiring precursors and cofactors from primary metabolism; hence, engineered primary metabolism is a prerequisite for biosynthesis of any secondary metabolite. 

One of the most important primary metabolic pathways is the oxidative PPP, which provides essential cofactors and intermediates for cell growth. The physiological effect of glucose-6-phosphate dehydrogenase (G6PDH) encoding isozymes in the PPP has been investigated in a variety of bacteria. Greater oxytetracycline (OTC) production is achieved by increasing the pool of malonyl-CoA, an OTC precursor, by deleting the *zwf1* and *zwf2 *genes encoding for two G6PDH isozymes of *S. rimosus *M4018 [22, 23]. However, the case was the opposite with 16.5% increase in daptomycin concentration when *zwf2* was overexpressed in *S. roseosporus* due to greater availability of daptomycin precursors by conversion to G6P in the PPP pathway. Deletion of *zwf1* or *zwf2* also improves actinorhodin and undecylprodigiosin production in *S. lividans* [24]. Significant changes in central carbon metabolism of *S. coelicolor* after deleting G6PDH and phosphoglucomutase (*pgm*) indicate that the carbon storage metabolism plays a significant role in precursor supply for actinorhodin production and overproduction [25]. These studies presumed that the lower flux of carbon through the PPP in each of the mutants allows for more efficient glucose utilization via glycolysis, resulting in higher levels of antibiotic production [24, 25]. Nevertheless, as G6PDH is the first enzyme in the PPP pathway and the key enzyme for generating NADPH, the increased biomass and NADPH regeneration would be another factor for favorable cell growth and precursor synthesis in both cases [26, 27]. Similarly, deleting the key glycolytic enzyme leads to a 2–6-fold enhancement of actinorhodin and undecylprodigiosin production in *S. coelicolor* A3 (2) on minimal and rich medim (2). The *pfkA2* (SCO5426, phosphofructokinase) deleted strain shows increased carbon flux through the PPP due to the accumulation of G6P and fructose-6-phosphate (F6P), which help to increase NADPH supply [28] and enhance antibiotic production as suggested by Gunnarsson et al. [26]. Supplementing the culture broth with a metabolic precursor to enhance poly-*ε*-lysine production by *S. noursei* NRRL 5126 is another robust example of an engineered metabolic network. Poly-*ε*-lysine is a strong inhibitor of a wide spectrum of microorganisms and is used as a food preservative. As an economically feasible process, supplementation with 5 mM citric acid and 2 mM L-aspartate increases *S. noursei* poly-*ε*-lysine production significantly in a time-dependent manner from 97.08 to 497.67 mg/L in 108 h [29]. FK506 (tacrolimus), a potent immunosuppressive agent, interacts with the FKBP12 receptor further targeting calcineurin by inhibiting Ser/Thr phosphatase activity leading to the arrest of T cell proliferation [30]. Engineering the pathway-specific building blocks for biosynthesis occasionally improves production of some polyketide or nonribosomal peptide natural products. For example, enhancing biosyntheses of methoxymalonyl-ACP and allylmalonyl-CoA, the extender units of FK506, together with optimizing glucose concentrations enhances the FK506 titer by approximately 150% in comparison to that of the original *S. tsukubaensis *strain [31]. Similarly, overexpressing potential biosynthetic sugar genes such as *desIII* (glucose-1-phosphate thymidylyl-transferase) and *desIV* (TDP-D-glucose 4,6-dehydratase) from *S. venezuelae* and glycosyltransferase DnrS*-DnrQ* transferring TDP-L-daunosamine to *ɛ*-rhodomycinone from *S. peucetius* leads to significantly enhanced doxorubicin (DXR) production [32]. These studies confirmed that engineering of rate limiting steps can be a robust strategy for efficient flow of intermediates to enhance production of secondary metabolites. 

## 3. Engineered Genomics

### 3.1. Genome Guided Overexpression of Gene Clusters in Native and Heterologous Hosts

Amplifiable units of DNA (AUDs) exist in *Streptomyces* with multiple copies per chromosome in tandem. They often lie in unstable chromosomal regions, such as ends of the linear chromosome, and a large deletion frequently accompanies amplification [33]. Consequently, inducible amplification of specific regions of microbial genomes helps to improve a wide variety of complex multigene processes in strains that biosynthesize various important secondary metabolites [34]. Yanai et al. [35] reported that amplifying the entire kanamycin (Km) biosynthetic gene cluster in *S. kanamyceticus* 12-6 results in a disparity in antibiotic production. A comparison of Km production from 12-6 (containing an average of three copies of the Km gene cluster) and 12-6-4 (containing one copy of the Km gene cluster) indicated that the titer of strain 12-6 strain (376 *μ*g/mL) was about twice that of strain 12-6-4 (196 *μ*g/mL). Furthermore, integrating cosmid pMJ20-10-1 (containing the entire Km gene cluster) into strain 12-6-4 (results in two copies of the Km gene cluster) results in a lower Km titer, indicating that integration of the cosmid suppresses Km production. This finding supports the notion that Km production level depends on the gene cluster copy number and that introducing an extra copy of the biosynthetic gene cluster into a parent strain may be an effective approach to improve antibiotic production [35]. Furthermore, an extra copy of the nikkomycin (a competitive inhibitor of chitin synthase with fungicidal, insecticidal, and acaricidal activities) biosynthetic gene cluster (35 kb) into *S. ansochromogenes* 7100 leads to enhanced production of nikkomycins (880 mg/L, 4-fold nikkomycin X and 210 mg/L, 1.8-fold nikkomycin Z) in the resulting exconjugants compared with that of the parent strain (220 mg/L nikkomycin X and 120 mg/L nikkomycin Z) [36]. Similarly, engineering *S. coelicolor* utilizing the *oriT*-like recombination sites *RsA* and *RsB* and *ZouA*, a site-specific relaxase flanking actinorhodin gene cluster, results in 4–12 tandem copies of the complete gene cluster, averaging nine repeats per genome leading to a 20-fold increase in actinorhodin production [34]. Thus, amplification of the entire gene cluster has direct positive effects on enzymatic yield and precursor flow leading to enhanced secondary metabolite production.

Extensive focus on the genetics of *Streptomyces *has [7–10] revealed numerous silent gene clusters probably biosynthesizing unknown complex natural products [37]. As an alternative, heterologous expression of these gene clusters in a suitable strain is a technique of pivotal importance to exploit drug discovery programs [38]. Normally, heterologous expression is preferably carried out in fully sequenced strains, such as *S. coelicolor* A3 (2) [8], *S. avermitilis *[9], or *S. venezuelae *(unpublished data, our group) due to unrestricted metabolic engineering for proper flux of precursors and regulatory networks. Furthermore, active secondary metabolic gene clusters of such strains may be silent to prevent diversion of precursors into competing secondary metabolic pathways, thus, facilitating enhanced production of the desired compounds [38]. A genetically engineered “clean” host strain, *S. coelicolor* CH999, has been constructed in which the entire actinorhodin gene cluster is surgically deleted [39]. The most intensively studied strains are *S. coelicolor* M145, M512, M1146, and M1154 [40–42], in which extensive deletion of different gene clusters has been performed to prevent background noise. A genome-minimized strain of *S. avermitilis* represents a suitable host for efficient production of secondary metabolites, as demonstrated by heterologous expression of the antibiotics streptomycin, cephamycin C, and pladienolide [43]. *S. venezuelae* YJ003, bearing a deletion of the pikromycin gene cluster, is also a widely used strain in our lab with the advantages of fast growth, good transformation efficiency, and rapid production of tylosin, kanamycin, spectinomycin, spectinamine, gentamicin, and epothilones (unpublished data) [44–47]. Similarly, other strains of *Streptomyces*, such as *S. lividans* TK23, TK24, and TK63, are also used as heterologous hosts to produce daptomycin and paromamine [48, 49]. A plasmid cured strain of *S. clavuligerus* has also been suggested as a heterologous host for secondary metabolites [50]. The heterologous expression of cryptic pathways in heterologous hosts suitable for expressing otherwise silent secondary metabolite gene clusters opens new avenues for the production of secondary metabolites that are not produced under normal laboratory conditions in native hosts. For example, capreomycin was produced at 50 mg/L from *S. lividans* without any modifications, whereas it was produced less by the native strain of *Saccharothrix mutabilis* and was not amenable to genetic studies [51]. In contrast, the cryptic gene cluster encoding thiocoraline biosynthesis from a marine *Micromonospora* sp. ML1 produces a significant amount of thiocoraline in *S. albus* J1074 [52]. Thus, a rationale approach for addressing the expression of cryptic pathways unexpressed or repressed in native hosts includes identifying novel pathways by bioinformatics and cloning and expressing them in well-characterized hosts with known secondary metabolomics [53].

### 3.2. Genome Shuffling Guided Enhancement of Secondary Metabolites

Genome shuffling is an amalgamation of classical breeding with modern high-throughput screening based on genome recombination even in the absence of detailed genetic knowledge [54, 55]. Genome shuffling combines the advantage of multiparental crossing facilitated by recombination of an entire genome associated with conventional breeding applications and, thus, acts as a combined method to improve phenotype [56]. Genome shuffling is a novel and promising technique discovered to enhance secondary metabolite production [57, 58]. Desired phenotypes can be obtained using this technique after several rounds of genome recombination of key genes responsible for production [54]. Genome shuffling basically incorporates (1) construction of diverse parental strains in several rounds of mutagenesis using chemical agents such as ethyl methanesulfonate and nitrosoguanidine as well as physical agents such as ultraviolet and *γ* irradiations; (2) recursive protoplast fusion of mutants with a multitude of phenotypes, and (3) intensive screening and selection based on product yields or other desired characteristics [59, 60]. 

Genome shuffling has been widely used to enhance secondary metabolite production in streptomycetes. Its first use was reported in *S. fradiae,* where significant phenotypic improvement was observed in just two rounds of genome shuffling [58]. Sixfold higher tylosin production was achieved from a hybrid strain, which was equivalent to achieving 20 rounds of classical strain improvement by random mutation that would probably require 20 years [61]. Nevertheless, production of about 3.5 g/L natamycin has been reported from *S. gilvosporeus *SG1, which was 153% of that of the parental strain and 1.17 times greater than that of the starting strain [62]. Luo et al. [63] also reported similar results, in which 4.7 g/L natamycin was produced in a shaking flask after a 96 h culture. This was 97.1% and 379% of the amount produced by the highest producing parental strain and the initial strain, respectively, after four rounds of genome shuffling.

*S. pristinaespiralis* produces pristinamycin (Ptr), an active drug against various multidrug-resistant pathogenic strains [64–67]. However, pristinamycin itself inhibits biosynthesis and mycelial growth [68]. Although antibiotic-producing streptomycetes have developed mechanisms to protect themselves against their own antibiotics, many antibiotics are toxic at elevated concentrations. This toxicity could be particularly problematic in the quest for antibiotic overproducing strains. However, it is not surprising that increased antibiotic resistance has often been used to select for mutants with increased antibiotic production levels. As a consequence, genome shuffling was used for *S. pristinaespiralis* to increase the resistivity against its own product from 20 to 100 *μ*g/mL, and production was increased from 0.47 g/L to 0.89 g/L after four rounds of shuffling [54]. A quantitative real-time polymerase chain reaction (qRT-PCR) analysis by Jin et al. [69] revealed the involvement of *snbA *and* snaB* (encoding the two subunits of pristinamycin II_A_ synthase catalyzing the last step in the biosynthesis of the pristinamycin II_A_ component) with higher expression in the recombinant than that in the ancestor at 24–60 h of fermentation, indicating that their expression changes might be a key factor during antibiotic biosynthesis. Similarly, the *ptr *resistance gene maintains a high expression level during the entire fermentation process of the recombinant strain, whereas it is expressed at a low level at 24–48 h of fermentation in the ancestor. These results indicate that the discrepancy in expression changes might be a key factor during antibiotic biosynthesis [69]. Similarly, amplified fragment length polymorphism analysis of a high-pristinamycin-producing strain revealed that a homolog of the *afsR* regulatory gene, a global regulator of secondary metabolism in *S. coelicolor* A3 (2) [40], and a homolog of the transposase gene, belonging to the validamycin biosynthetic gene cluster from *S. hygroscopicus* [70], are responsible for yield improvement in *S. pristinaespiralis *[69]. Similarly, genome shuffling of *Streptomyces *sp. U121, the producer of (2S, 3R)-HCA [71, 72] with potent pancreatic *α*-amylase and intestinal *α*-glucosidase inhibitory activities [73, 74], has been targeted to achieve rapid improvement in HCA production using the resistance mechanism for transepoxyaconitic acid (antibiotic HCA analog), resulting in fivefold higher HCA production than that in the wildtype after three rounds of shuffling [75]. This technique has also been suggested to increase *ε*-poly-l-lysine productivity in wildtype strains of *S. padanus, S. griseofuscus, S. graminearus, S. hygroscopicus, *and* S. albulus* through glucose tolerance, sulfaguanidine tolerance, and using succinic acid as the sole carbon source, respectively [76, 77]. Furthermore, *S. rimosus* was also subjected to genome shuffling for higher oxytetracycline yielding strains [60]. In combination with genome shuffling, ribosome engineering has been used in *S. viridochromogenes*, an avilamycin producer and an effective antimicrobial agent against multidrug-resistant Gram-positive bacteria [59]. A mutant strain obtained after *γ*-irradiation was used for genome shuffling with ribosome engineering mediated by streptomycin resistance. After five rounds of genome shuffling, 300 *μ*g/mL streptomycin resisting strain produced 1.4 g/L avilamycin, which was 4.8-fold and 36.8-fold greater than the shuffling starter and ancestor, respectively [59].

## 4. Engineered Proteomics

### 4.1. Proteomics Facilitates Reverse Engineering to Enhance Secondary Metabolite Production

Since the 1970s, recombinant DNA technology has revolutionized the ability to engineer microorganisms by modifying specific genes and pathways for optimized production of commercially significant metabolites [78]. In contrast, the concept of reverse engineering has evolved as a powerful tool in which two processes are utilized to genetically characterize existing overproducing strains, and a second generation of information is used for more efficient engineering of new strains that synthesize high yields of natural products [79]. This approach elucidates the interrelationships between physiological traits and more efficiently directs the engineering of target compound producing strains to synthesize high yields of these natural products [80]. For example, reverse engineering of the *S. coelicolor* overproducer using two-dimensional (2D) gel electrophoresis recently identified S-adenosyl methionine (SAM) synthetase as an antibiotic overproducing enzyme [81, 82]. Based on this observation, expression of the *metK* gene encoding SAM synthetase has been utilized to enhance pikromycin by 1.6-fold in *S. venezuelae *[83]. Similarly, exogenous feeding of SAM results in enhanced spectinomycin production by 3.6, and 3-fold in *S. griseus *IFO13189 using synthetic and nutrient media, respectively, and a 2-fold increase in bicozamycin production from *S. griseoflavus *FERM1805 [84]. Coexpression and standalone expression of *metK* or exogenous feeding of SAM results in enhanced antibiotic production in various streptomycetes, such as candicin D from *Streptomyces *sp. FR008 [85], avermectin from *S. avermitilis* [85], actinorhodin from *S. coelicolor *A3 (2) [82], actinorhodin from *S. lividans* TK23 [86], and oleandomycin from *S. antibioticus *ATCC11891 [87]. Furthermore, Zhuo et al. [80] also implemented the same technique to increase the production of avermectin in *S. avermitilis *in a round ofmicroarray studies confirming the overexpression of the pathway specific regulatory gene *aveR* in a high-producing strain. Based on the assumption that the promoter region of the *aveR* gene is recognized by sigma factor *σ* ^*hrdB*^, a mutant library of the *hrdB* gene was generated and overexpressed resulting in >50% improvement in avermectin B1 [80]. This example suggests that manipulating important genes revealed by reverse engineering can effectively improve the yield of target metabolites.

Advances in proteomics have made it possible to identify proteins that show significant changes in expression levels on 2D gel electrophoresis under certain conditions [88]. These approaches can also be used for engineering secondary metabolite target genes to enhance antibiotic production. For example, an attempt was made to increase the coenzyme A (CoA) pool using the pantothenate kinase (*panK*) gene to enhance production of DXR in *S. peucetius* ATCC 27952; however, the opposite occurred due to increased aglycone polyketide *ε*-rhodomycinone (RHO) [89]. To understand these results in detail, 2D gel electrophoresis was used to show that the efflux protein DrrA was overexpressed, resulting in 9.4-fold higher DXR production than that of a *panK* integrated strain, which showed that the proteomic approach is quite useful for host development and understanding the physiology of antibiotic production.

### 4.2. Ribosome Engineering to Enhance Secondary Metabolite Production

Ribosomes are the fundamental organelles controlling the protein-RNA complex expression machinery that synthesizes proteins using genetic instructions encoded in the mRNA template. Hence, engineering ribosomes to fine tune protein expression and secondary metabolite production is a highly utilized approach. One conventional method to modulate ribosomes is to introduce mutations conferring resistance to drugs that attack ribosomes, which frequently have mutations within ribosomal components (ribosomal protein, rRNA, or translation factors) [90–92]. For example, generating a point mutation in the ribosomal protein *rpsL *(str-6) of the *S. lividans* TK24 strain against *Streptomycin* (Str) causes production of the pigmented antibiotic actinorhodin, which is not produced under normal laboratory conditions, which could be due to significant changes in translational machinery [82]. Nearly half of the *str* mutants in *S. chattanoogensis* exhibit a significant increase in fredericamycin production (>five-fold), with one strain showing 26-fold higher antibiotic production than that of the wild type [91]. The frequency of such antibiotic overproducing strains among the *str* mutants is 3–46%, as shown with several strains in the genera *Streptomyces, Bacillus, and Pseudomonas* [91]. Moreover, biosynthesis of actinorhodin, undecylprodigiosin, and calcium-dependent antibiotics is markedly activated by introducing specific types of rifampicin (Rif) mutations into the *rpoB* (encoding the RNA polymerase subunit) gene in *S. lividans* 66 [93]. Furthermore, generating double mutants using gentamicin (Gen) or *rif* in str mutant further increases actinorhodin production by 1.7–2.5-fold, whereas triple mutants (*str, rif* and *gen*) produce almost 48 times more actinorhodin than that of the wild-type strain of *S. coelicolor* A3 (2) [94] and 2.3-fold higher salinomycin (10 mg/mL) in *S. albus *[95]. These single, double, and triple mutants display in hierarchical order a remarkable increase in the production of *act*II-ORF4, a pathway-specific regulatory protein that increases actinorhodin production [94]. Similarly, mutations conferring resistance to geneticin, fusidic acid, thiostrepton, and lincomycin generate quintuple, sextuple, septuple, and octuple mutants (C5, C6, C7, and C8, resp.) that produce 1.63 g/L and 1.22 g/L actinorhodin, which is 180, and 136-fold higher than that of the wild-type strain *S. coelicolor* A3 (2) in GYM33 media [96]. This dramatic overproduction of valuable drugs was the reason that ribosomal mutations and increased accumulation of bacterial alarmone (ppGpp) were found to play a pivotal role in the onset of antibiotic production in bacteria. Nevertheless, mutations in the RNA polymerase beta-subunit circumvent dependence on ppGpp production or increase stability of the 70S complex resulting in a higher translation level [95–97] and overproduction. However, the fundamental mechanism by which ribosomal engineering affects antibiotic production has been summarized in earlier reviews [98, 99]. 

Boosting translation during the stationary phase is another way to enhance secondary metabolite production in streptomycetes. Enhanced expression of the *frr* gene by mutations in the *rpsL* gene, which encodes a ribosome recycling factor (RRF), results in greater production of actinorhodin due to enhanced *S. coelicolor* protein synthesis [100], whereas overexpression of the *frr* gene increases avermectin yield (by 3 to 3.7-fold) in *S. avermitilis* strains due to the “copy number effect” of the *frr* gene [101]. Moreover, introducing *rpsL* and *rpoB* mutations in *S. coelicolor* enhances production of chloramphenicol and congocidine by 40-fold and 30-fold, respectively [42]. Furthermore, mutations in *rsmG* gene encoding for 16S rRNA methyltransferase [102] eventually lead to increase of the intracellular pool of SAM [103] and overproduction of antibiotics in streptomycetes[81, 82]. A combination of ribosomal engineering and reporter guided mutant selection helped to generate a daptomycin overproducing strain that produces twice as much A21978C (acidic cyclic lipopeptide antibiotic) as that of the parental strain of *S. roseosporus* [104].

## 5. Engineered Transcriptomics

Many successful stories of genome sequencing have been generated by efficient mining of genome data either *in silico* or in wet lab experiments. Several tools have been developed for functional studies of the basic unit of life; however, transcriptomic analysis was developed using microarray chip technology and mutational analysis and focuses on identifying the genes/regulators/regulons involved in the growth phase transition from primary to secondary metabolism in *S. coelicolor* [105]. Since then, immense interest has developed in transcriptome profiling of various *Streptomyces, *and studies have concluded that the expression of antibiotic biosynthetic genes is tightly controlled through multiple regulatory networks [106, 107]. Exploring the role of *aveI* (negative regulator) by microarray in combination with real-time reverse transcription PCR in *S. avermitilis* not only showed a negative effect on the avermectin biosynthetic gene cluster but also affected expression of the oligomycin and filipin biosynthetic clusters. In addition, the genes involved in precursor biosyntheses for avermectin or other antibiotics, such as crotonyl-CoA reductase and methylmalonyl-CoA decarboxylase, were also upregulated in the *aveI* mutant. Genes of several key primary metabolic pathways were downregulated in the mutant, suggesting that the *aveI* gene may function as a global regulator involved in directing carbon flux from primary to secondary metabolism [108]. Similarly, comparative transcriptome analysis between the low and high producer *S. avermitilis* using a whole-genome chip revealed the *tetR* family transcriptional regulator as a global upregulator for enhanced antibiotic production in *Streptomyces* species [109]. Moreover, the *wblA* gene is a pleiotropic downregulator of antibiotic biosynthesis in *Streptomyces* species based on a transcriptomics study using DNA microarray analysis for analyzing the discrepancy in mRNA abundance associated with DXR in *S. peucetiu*s overproducing industrial mutant (OIM) [100]. Furthermore, disruption of *wblA* from the *S. peucetius* OIM resulted in an additional 1.7-fold increase in the production of both DXR and daunorubicin (DNR) [107]. These results suggest that transcriptome based studies provide a comparative profile of gene expression at the molecular level and help to assess the key regulators for manifesting designer strains with enhanced secondary metabolite production. 

Recent “-omics” guided reverse engineering approaches, including comparative transcriptomics and proteomics, have been successfully used to identify alterations in gene expression associated with overproduction of secondary metabolites in industrial *Streptomyces* strains [79, 110–113]. The strategy to “reverse engineer” a reference organism (with a desirable property such as higher yield) is carried out by identifying the genetic or molecular basis of the property and subsequently reengineering the property into target organisms of interest by considering the key genes involved in complex mechanisms controlling microbial metabolism [80]. Successful reverse engineering depends on reproducibility of the overproducing mechanism in new target strains by using organisms whose genomic information is already available. An overproduction mutation is identified and a similar genetic manipulation is introduced into the same or closely related species, which is useful for achieving higher product titers without additional knowledge of concrete overproduction mechanisms [79]. New microarray and proteomic tools [105, 114, 115] as well as new tools for mutagenesis and mutant construction [116–118] are handy to characterize overproducing strains and thus bioengineer new target organisms for enhanced production. 

Similarly, precision engineering is a new approach that has been investigated to optimize existing biotechnology processes to improve desirable cell properties [119]. Askenazi et al. [120] described an approach to decipher the complex interrelationships between metabolite production and gene expression events to develop improved production strains. These advancements include transcriptional profiling using DNA microarrays, proteome profiling by 2D gel electrophoresis, and metabolite profiling by high-performance liquid chromatography. The cumulative information from these sources enables a more precise identification of key genetic targets and pathways engineered for strain improvement [121]. Single “-omic” analyses are not sufficient to fully unravel the complexities of microbial physiology and molecular biology associated with the production of secondary metabolites; thus, integrating different layers of information that is, multi “-omics” approaches, is essential to acquire precise insight into microorganisms and the mechanism of secondary metabolite overproduction. Thus, transcriptomics enables quantitative measurements of dynamic mRNA expression and variations between different states, reflecting the genes that are being overexpressed or downregulated at particular times and conditions. Hence, knowledge of transcriptomics is crucial for designing a rational integrated approach to enhance secondary metabolite production.

## 6. Future Perspectives

 Secondary metabolite production by streptomycetes can be efficiently enhanced by a number of approaches described herein; it is reasonable to expect that these techniques cannot be the endpoint. There are many such techniques developed for other bacterial genera to enhance secondary metabolite production, as engineered microbes typically require a high level of genetically stable expressing heterologous genes and pathways for genetic stability. For example, Tyo et al. developed a technique called chemically inducible chromosomal evolution (CIChE), which is a plasmid-free system for engineering *E. coli *with reduced allele segregation and enables roughly 2 to 4-fold increases in the yields of lycopene and the polymer poly-3-hydroxybutyrate [122, 123]. Similarly, reducing the number of plasmids to overcome differential gene expression by assembling a large construct from small fragments is becoming a popular technology in synthetic biology [124]. Manipulating gene clusters into monocistronic or pseudooperons has led to engineered biosynthesis of many natural products [125–128]. A tandemly placed repetitive promoter is another powerful technique for gene overexpression and enhanced metabolite production [129]. Strain development is still hampered by the intrinsic inefficiency of metabolic reactions caused by simple diffusion and random collisions of enzymes and metabolites. A scaffold system, which promotes the proximity of metabolic enzymes and increases the local concentration of intermediates, is a promising solution for this problem because scaffolds help to (1) increase the local concentration of intermediates around the enzymes on the scaffold, (2) prevent the loss of intermediates by diffusion or by competing reactions and (3) overcome feedback inhibition on other pathways due to the rapid conversion of feedback inhibitors [130–132]. Several successful examples of “-omics” technologies for drug production have already appeared, and this trend will continue at an accelerated pace. It is expected that microbial metabolic engineering will become an essential platform for developing and producing drugs in the near future.

## Figures and Tables

**Table 1:** List of bioactive compounds produced by streptomycetes.

Drugs	Strains	Applications
Aclacinomycin A	*S. galilaeus *	Antitumor
Actinorhodin	*S. coelicolor *	Antibacterial
Alnumycin	*Streptomyces* sp. CM020	Antitumor; gyrase inhibitor; topoisomerase inhibitor
Alpha-lipomycin	*S. aureofaciens *	Antibacterial
Amphotericin B	*S. nodosus *	Antifungal
Apramycin	*S. tenebrarius *	Antibacterial
Aranciamycin	*S. echinatus *	Antibacterial; Collagenase inhibitor
Ascomycin	*S. hygroscopicus *var.* ascomyceticus *	Immunosuppressive; antifungal
Asukamycin	*S. nodosus* subsp. *asukaensis *	Antitumor
Aureothin	*S. thioluteus *	Antitumor; antifungal; insecticidal
Avermectin	*S. avermitilis *	Anthelmintic
Benastatin	*Streptomyces* sp. A2991200	Antibacterial; apoptosis inducer; glutathione-S-transferase (GST) inhibitor
Bleomycin	*S.verticillus *	Antitumor
Borrelidin	*S. parvulus* Tü 4055	Angiogenesis inhibitor; antibacterial; antiviral; Antiproliferative
Chalcomycin	*S. bikiniensis *	Antibacterial
Chartreusin	*S. chartreusis *	Antibacterial; antitumor
Chlorothricin	*S. antibioticus *	Antibacterial
Chloramphenicol	*S. venezuelae *	Antibacterial
Chromomycin	*S. griseus *	Antibacterial; antitumor; antiviral
Coumermycin	*S. rishiriensis *	Antibacterial
Concanamycin A	*S. neyagawaensis *	Antifungal; antiprotozoal; antitumor; antiviral
Clavulanic acid	*S. clavuligerus *	Antibacterial
Cosmomycin	*S. olindensis *	Antitumor
Daptomycin	*S. roseosporus *	Antibacterial
Daunorubicin	*S. peucetius* subsp. *caesius *	Antitumor
Doxorubicin	*S. peucetius* subsp. *caesius *	Antitumor
Dunaimycins	*S. diastatochromogenes *	Immunosuppressive
Elloramycin	*S. olivaceus *	Antibacterial; antitumor
Enterocin	*S. maritimus *	Antibacterial
Formycin	*S. lavendulae *	Antitumor
Fredericamycin	*S. griseus; S. chattanoogensis *	Antibacterial; antifungal; antitumor
Frenolicin	*S. roseofulvus *	Antibacterial; antiprotozoal
Gilvocarcin	*S. griseoflavus; S. anandii *	Antibacterial; antitumor; antiviral
Granaticin	*S. violaceoruber *	Antibacterial
Griseorhodin A	*Streptomyces* sp. JP95	Antibacterial; human telomerase inhibitor
Geldanamycin	*S. hygroscopicus *	Antitumor
Griseusin	*S. griseus *	Antibacterial
Halstoctacosanolide	*S. halstedii *	Antibacterial
Hedamycin	*S. griseoruber *	Antitumor
Herbimycin A	*S. hygroscopicus *	Antitumor
Herboxidiene	*S. chromofuscus *	Herbicidal
Hygromycin B	*S. hygroscopicus *	Antibacterial; antifungal
Indanomycin	*S. antibioticus *	Antibacterial; antiprotozoal; insecticidal; Ionophore
Istamycin	*S. tenjimariensis *	Antibacterial
Jadomycin B	*S. venezuelae *	Antibacterial
Kanamycin	*S. kanamyceticus *	Antibacterial
Kirromycin	*S. collinus *	Antibacterial
Landomycin	*S. cyanogenus *	Antitumor
Lasalocid	*S. lasaliensis *	Antibacterial
Lymphostin	*Streptomyces* sp. KY11783	Immunosuppressive
Medermycin	*Streptomyces* sp. AM7161	Antibacterial; antitumor
Meilingmycin	*S. nanchangensis *	Anthelmintic
Meridamycin	*S. violaceusniger *	Neuroprotective
Mitomycin C	*S. caespitosus *	Antibacterial; antineoplastic; immunosuppressive
Mithramycin	*S. argillaceus *	Antibacterial; antitumor
Monensin	*S. cinnamonensis *	Antibacterial; ionophore; antiprotozoal
Nanchangmycin	*S. nanchangensis *	Antibacterial; insecticidal; ionophore
Naphthocyclinone	*S. arenae *	Antibacterial
Neomycin	*S. faradae *	Antibacterial
Niddamycin	*S. caelestis *	Antibacterial
Neocarzinostatin	*S. carzinostaticus *	Antibacteria; antineoplastic
NFAT-133	*Streptomyces* sp. PM324667	Antidiabetic
Nigericin	*S. violaceusniger *	Antibacterial; ionophore
Nogalamycin	*S. nogalater *	Antibacterial; antitumor
Nikkomycin X	*S. ansochromogenes *	Antibacterial
Nystatin	*S. noursei *	Antifungal
Novobiocin	*S. niveus *	Antibacterial
Oligomycin	*S. avermitilis *	Antifungal
Oviedomycin	*S. antibioticus *	Antitumor
Oxazolomycin	*S. albus *	Antibacterial; antitumor; antivirus; ionophore
Oxytetracycline	*S. rimosus *	Antibacterial
Pactamycin	*S. pactum *	Antibacterial; antiprotozoal; antitumor; antiviral
Paromycin	*S. rimosus *	Antiamoebal, antibiotics
Phoslactomycin	*Streptomyces* sp. HK803	Antitumor
Pikromycin	*S. venezuelae *	Antibacterial
Pimaricin	*S. natalensis *	Antifungal
Pladienolide	*S. platensis *	Antitumor
Pristinamycin	*S. pristinaespiralis *	Antibacterial
Polyketomycin	*S. diastatochromogenes *	Antibacterial; antitumor
Pikromycin	*S. venezuelae *	Antibacterial
Rapamycin	*S. hygroscopicus *	Immunosuppressive; antitumor; neuroprotective; antiaging
Ribostamycin	*S. ribosidificus *	Antibacterial
Resistomycin	*S. resistomycificus *	Antibacterial; antiviral
Rimocidin	*S. diastaticus *	Antifungal
Rubradirin	*S. achromogenes* var. *rubradiris *	Antibacterial
Saframycin A	*S. lavendulae *	Antitumor
Steffimycin	*S. steffisburgensis *	Antitumor
Streptolydigin	*S. lydicus *	Antibacterial
Sparsomycin	*S. sparsogenes *	Antitumor
Spiramycin	*S. ambofaciens. *	Antibacterial
Spectinomycin	*S. spectabilis *	Antibacterial
Streptomycin	*S. greseus *	Antibacterial
Tautomycetin	*Streptomyces* sp. CK4412	Antifungal; antitumor; immunosuppressive
Tautomycin	*S. spiroverticillatus *	Antibacterial; antifungal; antitumor
Tetronomycin	*Streptomyces* sp. NRRL11266	Antibacterial; ionophore
Thiostrepton	*S. azureus *	Antibacterial
Tetracycline	*S. aureofaciens *	Antibacterial
Urdamycin	*S. fradiae *	Antibacterial; antitumor
Vicenistatin	*S. halstedii *	Antitumor
Virginiamycin	*S. virginiae *	Antibacterial

**Table 2:** Methodologies used to overproduce drugs using engineered metabolic networks approach.

Strains	Drugs	Approach	Methodologies
*S. rimosus *M4018	Oxytetracycline	Engineered metabolic networks	Deletion of *zwf1* and *zwf2* genes improve the production of oxytetracycline
*S. roseosporus *	Daptomycin	Engineered metabolic networks	Over-expression of zwf2 gene improve the production of daptomycin
*S. lividans *	Actinorhodin and undecylprodigiosin	Engineered metabolic networks	Deletion of *zwf1* or *zwf2* improved actinorhodin and undecylprodigiosin production
*S. coelicolor* A3 (2)	Actinorhodin and undecylprodigiosin	Engineered metabolic networks	Deletion of *pfkA2* (phosphofructokinase) gene improve the production of actinorhodin and undecylprodigiosin
*S. noursei* NRRL 5126	*ε*-Poly-l-Lysine	Engineered metabolic networks	Supplementation of citric acid and L-Asp increases poly-*ε*-lysine production
*S. tsukubaensis *	FK506 (tacrolimus)	Engineered metabolic networks	Enhancing the biosyntheses of methoxymalonyl-ACP and allylmalonyl-CoA together with optimized glucose concentrations enhances the FK506 production
*S. peucetius* ATCC 27952	Doxorubicin	Engineered metabolic networks	Over-expression of potential biosynthetic sugar genes and glycosyltransferase enhanced doxorubicin production

**Table 3:** Methodologies used to overproduce drugs using engineered genomics approach.

Strains	Drugs	Approach	Methodologies
*S. kanamyceticus *	Kanamycin	Genome guided overexpression of gene clusters in native and heterologous hosts	Overexpression of extra copy of the gene cluster enhanced kanamycin production
*S. ansochromogenes *	Nikkomycin	Genome guided overexpression of gene clusters in native and heterologous hosts	Overexpression of extra copy of the gene cluster enhanced nikkomycins production
*S. coelicolor *	Actinorhodin	Genome guided overexpression of gene clusters in native and heterologous hosts	Tandem copies of the gene cluster increased actinorhodin production
*S. avermitilis *	Streptomycin, cephamycin C, and pladienolide	Genome guided overexpression of gene clusters in native and heterologous hosts	Heterologous expression in genome-minimized strain
*S. venezuelae* YJ003	Tylosin, kanamycin, spectinomycin, spectinamine, gentamicin, and epothilones	Genome guided overexpression of gene clusters in native and heterologous hosts	Heterologous expression in pikromycin gene cluster deleted strain
*S. lividans* TK-23, TK-24, and TK-63	Daptomycin and paromamine	Genome guided overexpression of gene clusters in native and heterologous hosts	Heterologous expression
*S. lividans *	Capreomycin	Genome guided overexpression of gene clusters in native and heterologous hosts	Heterologous expression
*S. albus* J1074	Thiocoraline	Genome guided overexpression of gene clusters in native and heterologous hosts	Heterologous expression
*S. fradiae *	Tylosin	Genome shuffling guided enhancement of secondary metabolites	Two rounds of genome shuffling
*S. gilvosporeus *SG1	Natamycin	Genome shuffling guided enhancement of secondary metabolites	Four rounds of genome shuffling
*S. pristinaespiralis *	Pristinamycin	Genome shuffling guided enhancement of secondary metabolites	Four rounds of genome shuffling to increase the resistivity against pristinamycin enhanced pristinamycin production
*S. *sp. U121	(2S, 3R)-HCA	Genome shuffling guided enhancement of secondary metabolites	Generating resistance mechanism for transepoxyaconitic acid by three rounds of shuffling
*S. padanus,* *S. griseofuscus,* *S. graminearus,* *S. hygroscopicus,* and *S. albulus *	*ε*-Poly-l-lysine	Genome shuffling guided enhancement of secondary metabolites	Through glucose, sulfa guanidine, and succinic acid tolerance and genome shuffling

**Table 4:** Methodologies used to overproduce drugs using engineered proteomics approach.

Strains	Drugs	Approach	Methodologies
*S. venezuelae *	Pikromycin	Proteomics facilitated reverse engineering to enhance secondary metabolite production	Overexpression of the metK gene encoding SAM synthetase
*S. griseus *IFO13189 and *S. griseoflavus *FERM1805	Spectinomycin and bicozamycin	Proteomics facilitated reverse engineering to enhance secondary metabolite production	Exogenous feeding of S-Adenosyl methionine (SAM) results in enhanced production
*S*. sp. FR-008, *S. avermitilis, S. coelicolor *A3 (2), *S. lividans* TK23, and *S. antibioticus *ATCC11891	Candicin D, avermectin, actinorhodin, and oleandomycin	Proteomics facilitated reverse engineering to enhance secondary metabolite production	Coexpression of metK or exogenous feeding of SAM enhanced antibiotic production
*S. avermitilis *	Avermectins	Proteomics facilitated reverse engineering to enhance secondary metabolite production	Overexpression of mutant library of sigma factor σ^hrdB^ enhanced antibiotic production
*S. peucetius* ATCC 27952	Doxorubicin	Proteomics facilitated reverse engineering to enhance secondary metabolite production	Overexpression of efflux protein DrrA enhanced antibiotic production
*S. lividans* TK24	Actinorhodin	Ribosome engineering to enhance secondary metabolite production	Resistance against streptomycin causes production of pigmented antibiotic actinorhodin not produced in normal laboratory conditions
*S. chattanoogensis *	Fredericamycin	Ribosome engineering to enhance secondary metabolite production	Resistance against streptomycin causes enhanced production
*S. lividans* 66	Actinorhodin, undecylprodigiosin, and calcium dependent antibiotics	Ribosome engineering to enhance secondary metabolite production	By introducing rifampicin mutations into the rpoB (encoding the RNA polymerase subunit) gene
*S. coelicolor* A3 (2)	Actinorhodin	Ribosome engineering to enhance secondary metabolite production	By introducing double and triple mutations using gentamicin rifampicin and streptomycin increases actinorhodin production
*S. coelicolor* A3 (2)	Actinorhodin	Ribosome engineering to enhance secondary metabolite production	Enhanced expression of ribosome recycling factor by mutation increases production
*S. avermitilis *	Avermectin	Ribosome engineering to enhance secondary metabolite production	Overexpression of ribosome recycling factor increases production
*S. coelicolor* A3 (2)	Chloramphenicol and congocidine	Ribosome engineering to enhance secondary metabolite production	Introducing rpsL and rpoB mutations enhanced chloramphenicol and congocidine production
Streptomycetes	Antibiotics	Ribosome engineering to enhance secondary metabolite production	Introducing mutations in rsmG gene encoding for 16S rRNA methyltransferase

**Table 5:** Methodologies used to overproduce drugs using engineered transcriptomics approach.

Strains	Drugs	Approach	Methodologies
Streptomycetes	Antibiotics	Engineered Transcriptomics	TetR family transcriptional regulator as a global upregulator for enhanced antibiotic production
*S. peucetius* OIM	Doxorubicin and Daunorubicin	Engineered Transcriptomics	Disruption of wblA from S. peucetius OIM resulted in increase in the production of both doxorubicin and daunorubicin

## References

[1] Demain AL, Fang A (2000). The natural functions of secondary metabolites. *Advances in Biochemical Engineering/Biotechnology*.

[2] Bérdy J (2005). Bioactive microbial metabolites. *The Journal of Antibiotics*.

[3] Newman DJ, Cragg GM (2007). Natural products as sources of new drugs over the last 25 years. *Journal of Natural Products*.

[4] Dufour N, Rao RP (2011). Secondary metabolites and other small molecules as intercellular pathogenic signals. *FEMS Microbiology Letters*.

[5] Waksman SA, Henrici AT (1943). The nomenclature and classification of the Actinomycetes. *Journal of Bacteriology*.

[6] Wildermuth H, Hopwood DA (1970). Septation during sporulation in *Streptomyces coelicolor*. *Journal of General Microbiology*.

[7] Omura S, Ikeda H, Ishikawa J (2001). Genome sequence of an industrial microorganism *Streptomyces avermitilis*: deducing the ability of producing secondary metabolites. *Proceedings of the National Academy of Sciences of the United States of America*.

[8] Bentley SD, Chater KF, Cerdeño-Tárraga A-M (2002). Complete genome sequence of the model actinomycete *Streptomyces coelicolor* A3(2). *Nature*.

[9] Ikeda H, Ishikawa J, Hanamoto A (2003). Complete genome sequence and comparative analysis of the industrial microorganism *Streptomyces avermitilis*. *Nature Biotechnology*.

[10] Ohnishi Y, Ishikawa J, Hara H (2008). Genome sequence of the streptomycin-producing microorganism *Streptomyces griseus* IFO 13350. *Journal of Bacteriology*.

[11] Nett M, Ikeda H, Moore BS (2009). Genomic basis for natural product biosynthetic diversity in the actinomycetes. *Natural Product Reports*.

[12] Hopwood DA (2006). Soil to genomics: the *Streptomyces* chromosome. *Annual Review of Genetics*.

[13] Liebler DC (2002). *Introduction to Proteomics: Tools for the New Biology*.

[14] Wilkins MR, Appel RD, Williams KL, Hochstrasser DF (2007). *Proteome Research: Concepts, Technology and Application*.

[15] Nielsen J, Keasling JD (2011). Synergies between synthetic biology and metabolic engineering. *Nature Biotechnology*.

[16] Fleischmann RD, Adams MD, White O (1995). Whose-genome random sequencing and assembly of *Haemophilus influenzae* Rd. *Science*.

[17] Varma A, Palsson BO (1994). Stoichiometric flux balance models quantitatively predict growth and metabolic by-product secretion in wild-type *Escherichia coli* W3110. *Applied and Environmental Microbiology*.

[18] Hodgson DA (2000). Primary metabolism and its control in streptomycetes: a most unusual group of bacteria. *Advances in Microbial Physiology*.

[19] Bro C, Nielsen J (2004). Impact of “ome” analyses on inverse metabolic engineering. *Metabolic Engineering*.

[20] Nguyen Q-T, Merlo ME, Medema MH, Jankevics A, Breitling R, Takano E (2012). Metabolomics methods for the synthetic biology of secondary metabolism. *FEBS Letters*.

[21] Aharoni A, Galili G (2011). Metabolic engineering of the plant primary-secondary metabolism interface. *Current Opinion in Biotechnology*.

[22] Liu Z, Guo M, Qian J, Zhuang Y, Zhang S (2008). Disruption of zwf2 gene to improve oxytetraclyline biosynthesis in *Streptomyces rimosus* M4018. *Wei Sheng Wu Xue Bao*.

[23] Tang Z, Xiao C, Zhuang Y (2011). Improved oxytetracycline production in *Streptomyces rimosus* M4018 by metabolic engineering of the G6PDH gene in the pentose phosphate pathway. *Enzyme and Microbial Technology*.

[24] Butler MJ, Bruheim P, Jovetic S, Marinelli F, Postma PW, Bibb MJ (2002). Engineering of primary carbon metabolism for improved antibiotic production in *Streptomyces lividans*. *Applied and Environmental Microbiology*.

[25] Ryu Y-G, Butler MJ, Chater KF, Lee KJ (2006). Engineering of primary carbohydrate metabolism for increased production of actinorhodin in *Streptomyces codicolor*. *Applied and Environmental Microbiology*.

[26] Gunnarsson N, Eliasson A, Nielsen J (2004). Control of fluxes towards antibiotics and the role of primary metabolism in production of antibiotics. *Advances in Biochemical Engineering/Biotechnology*.

[27] Huang D, Wen J, Wang G, Yu G, Jia X, Chen Y (2012). In silico aided metabolic engineering of *Streptomyces roseosporus* for daptomycin yield improvement. *Applied Microbiology and Biotechnology*.

[28] Borodina I, Siebring J, Zhang J (2008). Antibiotic overproduction in *Streptomyces coelicolor* A3(2) mediated by phosphofructokinase deletion. *Journal of Biological Chemistry*.

[29] Bankar SB, Singhal RS (2011). Metabolic precursors enhance the production of poly-*ε*-lysine by *Streptomyces noursei* NRRL 5126. *Engineering in Life Sciences*.

[30] Liu J, Farmer JD, Lane WS, Friedman J, Weissman I, Schreiber SL (1991). Calcineurin is a common target of cyclophilin-cyclosporin A and FKBP-FK506 complexes. *Cell*.

[31] Chen D, Zhang Q, Zhang Q, Cen P, Xu Z, Liu W (2012). Improvement of FK506 production in *Streptomyces tsukubaensis* by genetic enhancement of the supply of unusual polyketide extender units viautilization of two distinct site-specific recombination systems. *Applied and Environmental Microbiology*.

[32] Malla S, Niraula NP, Liou K, Sohng JK (2009). Enhancement of doxorubicin production by expression of structural sugar biosynthesis and glycosyltransferase genes in *Streptomyces peucetius*. *Journal of Bioscience and Bioengineering*.

[33] Volff J-N, Altenbuchner J (1998). Genetic instability of the *streptomyces* chromosome. *Molecular Microbiology*.

[34] Murakami T, Burian J, Yanai K, Bibb MJ, Thompson CJ (2011). A system for the targeted amplification of bacterial gene clusters multiplies antibiotic yield in *Streptomyces coelicolor*. *Proceedings of the National Academy of Sciences of the United States of America*.

[35] Yanai K, Murakami T, Bibb M (2006). Amplification of the entire kanamycin biosynthetic gene cluster during empirical strain improvement of *Streptomyces kanamyceticus*. *Proceedings of the National Academy of Sciences of the United States of America*.

[36] Liao G, Li J, Li L, Yang H, Tian Y, Tan H (2010). Cloning, reassembling and integration of the entire nikkomycin biosynthetic gene cluster into *Streptomyces ansochromogenes* lead to an improved nikkomycin production. *Microbial Cell Factories*.

[37] Corre C, Challis GL (2009). New natural product biosynthetic chemistry discovered by genome mining. *Natural Product Reports*.

[38] Flinspach K, Westrich L, Kaysser L (2010). Heterologous expression of the biosynthetic gene clusters of coumermycina1, clorobiocin and caprazamycins in genetically modified *Streptomyces coelicolor* strains. *Biopolymers*.

[39] McDaniel R, Ebert-Khosla S, Hopwood DA, Khosla C (1993). Engineered biosynthesis of novel polyketides. *Science*.

[40] Floriano B, Bibb M (1996). afsR is a pleiotropic but conditionally required regulatory gene for antibiotic production in *Streptomyces coelicolor* A3(2). *Molecular Microbiology*.

[41] Hosaka T, Ohnishi-Kameyama M, Muramatsu H (2009). Antibacterial discovery in actinomycetes strains with mutations in RNA polymerase or ribosomal protein S12. *Nature Biotechnology*.

[42] Gomez-Escribano JP, Bibb MJ (2011). Engineering *Streptomyces coelicolor* for heterologous expression of secondary metabolite gene clusters. *Microbial Biotechnology*.

[43] Komatsu M, Uchiyama T, Omura S, Cane DE, Ikeda H (2010). Genome-minimized *Streptomyces* host for the heterologous expression of secondary metabolism. *Proceedings of the National Academy of Sciences of the United States of America*.

[44] Jung WS, Lee SK, Hong JSJ (2006). Heterologous expression of tylosin polyketide synthase and production of a hybrid bioactive macrolide in *Streptomyces venezuelae*. *Applied Microbiology and Biotechnology*.

[45] Thapa LP, Oh T-J, Lee HC (2007). Heterologous expression of the kanamycin biosynthetic gene cluster (pSKC2) in *Streptomyces venezuelae* YJ003. *Applied Microbiology and Biotechnology*.

[46] Je WP, Hong JSJ, Parajuli N (2008). Genetic dissection of the biosynthetic route to gentamicin A2 by heterologous expression of its minimal gene set. *Proceedings of the National Academy of Sciences of the United States of America*.

[47] Park SR, Park JW, Jung WS (2008). Heterologous production of epothilones B and D in *Streptomyces venezuelae*. *Applied Microbiology and Biotechnology*.

[48] Penn J, Li X, Whiting A (2006). Heterologous production of daptomycin in *Streptomyces lividans*. *Journal of Industrial Microbiology and Biotechnology*.

[49] Nepal KK, Oh T-J, Sohng JK (2009). Heterologous production of paromamine in *Streptomyces lividans* TK24 using kanamycin biosynthetic genes from *Streptomyces kanamyceticus* ATCC12853. *Molecules and Cells*.

[50] Medema MH, Trefzer A, Kovalchuk A (2010). The sequence of a 1.8-Mb bacterial linear plasmid reveals a rich evolutionary reservoir of secondary metabolic pathways. *Genome Biology and Evolution*.

[51] Felnagle EA, Rondon MR, Berti AD, Crosby HA, Thomas MG (2007). Identification of the biosynthetic gene cluster and an additional gene for resistance to the antituberculosis drug capreomycin. *Applied and Environmental Microbiology*.

[52] Lombó F, Velasco A, Castro A (2006). Deciphering the biosynthesis pathway of the antitumor thiocoraline from a marine actinomycete and its expression in two *Streptomyces* species. *ChemBioChem*.

[53] Baltz RH (2010). *Streptomyces* and *Saccharopolyspora* hosts for heterologous expression of secondary metabolite gene clusters. *Journal of Industrial Microbiology and Biotechnology*.

[54] Xu B, Jin Z, Wang H, Jin Q, Jin X, Cen P (2008). Evolution of *Streptomyces pristinaespiralis* for resistance and production of pristinamycin by genome shuffling. *Applied Microbiology and Biotechnology*.

[55] Jin ZH, Xu B, Lin SZ, Jin QC, Cen PL (2009). Enhanced production of spinosad in saccharopolyspora spinosa by genome shuffling. *Applied Biochemistry and Biotechnology*.

[56] Stephanopoulos G (2002). Metabolic engineering by genome shuffling. *Nature Biotechnology*.

[57] Patnaik R, Louie S, Gavrilovic V (2002). Genome shuffling of *Lactobacillus* for improved acid tolerance. *Nature Biotechnology*.

[58] Zhang Y-X, Perry K, Vinci VA, Powell K, Stemmer WPC, Del Cardayré SB (2002). Genome shuffling leads to rapid phenotypic improvement in bacteria. *Nature*.

[59] Lv XA, Jin YY, Li YD, Zhang H, Liang XL (2013). Genome shuffling of *Streptomyces viridochromogenes* for improved production of avilamycin. *Applied Microbiology and Biotechnology*.

[60] Zhitang L, Dawei Z, Zhi L, Ye L (2013). Nitrosoguanidine mutagenesis and genome shuffling enhanced the oxytetracycline production of *Streptomyces rimosus*. *IOSR Journal of Pharmacy and Biological Sciences*.

[61] Gong J, Zheng H, Wu Z, Chen T, Zhao X (2009). Genome shuffling: progress and applications for phenotype improvement. *Biotechnology Advances*.

[62] Zhu H, Jin Z-H, Cen P-L (2006). Natamycin-producing strain breeding by genome shuffling. *Chinese Journal of Antibiotics*.

[63] Luo JM, Li JS, Liu D (2012). Genome shuffling of *Streptomyces gilvosporeus* for improving natamycin production. *Journal of Agricultural and Food Chemistry*.

[64] Paquet V, Goma G, Soucaille P (1992). Induction of pristinamycins production in *Streptomyces pristinaespiralis*. *Biotechnology Letters*.

[65] Leclercq R, Soussy CJ, Weber P, Moniot-Ville N, Dib C (2003). In vitro activity of the pristinamycin against the isolated *Staphylococci* in the french hospitals in 1999-2000. *Pathologie Biologie*.

[66] Ng J, Gosbell IB (2005). Successful oral pristinamycin therapy for osteoarticular infections due to methicillin-resistant *Staphylococcus aureus* (MRSA) and other Staphylococcus spp. *Journal of Antimicrobial Chemotherapy*.

[67] Witte W, Naber KG, Pasemann B, Cuny C, Klare I (1996). In vitro sensitivity of *Staphylococci* against the pristinamycin combination RP59500. *Chemotherapie Journal*.

[68] Jia B, Jin Z-H, Lei Y-L, Mei L-H, Li N-H (2006). Improved production of pristinamycin coupled with an adsorbent resin in fermentation by *Streptomyces pristinaespiralis*. *Biotechnology Letters*.

[69] Jin Q, Jin Z, Zhang L, Yao S, Li F (2012). Probing the molecular mechanisms for pristinamycin yield enhancement in *Streptomyces pristinaespiralis*. *Current Microbiology*.

[70] Bai L, Li L, Xu H (2006). Functional analysis of the validamycin biosynthetic gene cluster and engineered production of validoxylamine A. *Chemistry and Biology*.

[71] Hida H, Yamada T, Yamada Y (2005). Production of hydroxycitric acid by microorganisms. *Bioscience, Biotechnology and Biochemistry*.

[72] Hida H, Yamada T, Yamada Y (2006). Absolute configuration of hydroxycitric acid produced by microorganisms. *Bioscience, Biotechnology and Biochemistry*.

[73] Hansawasdi C, Kawabata J, Kasai T (2000). *α*-amylase inhibitors from roselle (Hibiscus sabdariffa Linn.) tea. *Bioscience, Biotechnology and Biochemistry*.

[74] Yamada T, Hida H, Yamada Y (2007). Chemistry, physiological properties, and microbial production of hydroxycitric acid. *Applied Microbiology and Biotechnology*.

[75] Hida H, Yamada T, Yamada Y (2007). Genome shuffling of *Streptomyces* sp. U121 for improved production of hydroxycitric acid. *Applied Microbiology and Biotechnology*.

[76] Li S, Li F, Chen X-S (2012). Genome shuffling enhanced *ε*-poly-l-lysine production by improving glucose tolerance of *Streptomyces graminearus*. *Applied Biochemistry and Biotechnology*.

[77] Li S, Chen X, Dong C, Zhao F, Tang L, Mao Z (2013). Combining genome shuffling and interspecific hybridization among *Streptomyces* improved *ε*-Poly-l-Lysine production. *Applied Biochemistry and Biotechnology*.

[78] Crawford DL (1988). Development of recombinant *Streptomyces* for biotechnological and environmental uses. *Biotechnology Advances*.

[79] Lum AM, Huang J, Hutchinson CR, Kao CM (2004). Reverse engineering of industrial pharmaceutical-producing actinomycete strains using DNA microarrays. *Metabolic Engineering*.

[80] Zhuo Y, Zhang W, Chen D (2010). Reverse biological engineering of hrdB to enhance the production of avermectins in an industrial strain of *Streptomyces avermitilis*. *Proceedings of the National Academy of Sciences of the United States of America*.

[81] Kim D-J, Huh J-H, Yang Y-Y (2003). Accumulation of S-adenosyl-L-methionine enhances production of actinorhodin but inhibits sporulation in *Streptomyces lividans* TK23. *Journal of Bacteriology*.

[82] Okamoto S, Lezhava A, Hosaka T, Okamoto-Hosoya Y, Ochi K (2003). Enhanced expression of S-adenosylmethionine synthetase causes overproduction of actinorhodin in *Streptomyces coelicolor* A3(2). *Journal of Bacteriology*.

[83] Maharjan S, Oh T-J, Lee HC, Sohng JK (2008). Heterologous expression of metK1-sp and afsR-sp in *Streptomyces venezuelae* for the production of pikromycin. *Biotechnology Letters*.

[84] Saito N, Kurosawa K, Xu J, Okamoto S, Ochi K (2003). Effect of s-adenosylmethionine on antibiotic production in *Streptomyces griseus* and *Streptomyces griseoflavus*. *Actinomycetologica*.

[85] Wang T, Bai L, Zhu D (2012). Enhancing macrolide production in *Streptomyces* by coexpressing three heterologous genes. *Enzyme and Microbial Technology*.

[86] Zhang X, Fen M, Shi X, Bai L, Zhou P (2008). Overexpression of yeast S-adenosylmethionine synthetase metK in *Streptomyces actuosus* leads to increased production of nosiheptide. *Applied Microbiology and Biotechnology*.

[87] Zhao X-Q, Jin Y-Y, Kwon H-J, Yang Y-Y, Suh J-W (2006). S-Adenosylmethionine (SAM) regulates antibiotic biosynthesis in *Streptomyces* spp. in a mode independent of its role as a methyl donor. *Journal of Microbiology and Biotechnology*.

[88] Yates JR (2004). Mass spectral analysis in proteomics. *Annual Review of Biophysics and Biomolecular Structure*.

[89] Song E, Malla S, Yang Y-H (2011). Proteomic approach to enhance doxorubicin production in panK-integrated *Streptomyces peucetius* ATCC 27952. *Journal of Industrial Microbiology and Biotechnology*.

[90] Shima J, Hesketh A, Okamoto S, Kawamoto S, Ochi K (1996). Induction of actinorhodin production by rpsL (encoding ribosomal protein S12) mutations that confer streptomycin resistance in *Streptomyces lividans* and *Streptomyces coelicolor* A3(2). *Journal of Bacteriology*.

[91] Hosoya Y, Okamoto S, Muramatsu H, Ochi K (1998). Acquisition of certain streptomycin-resistant (str) mutations enhances antibiotic production in bacteria. *Antimicrobial Agents and Chemotherapy*.

[92] Okamoto-Hosoya Y, Sato T-A, Ochi K (2000). Resistance to paronomycin is conferred by rpsl. mutations, accompanied by an enhanced antibiotic production in *Streptomyces coelicolor* A3(2). *Journal of Antibiotics*.

[93] Hu H, Zhang Q, Ochi K (2002). Activation of antibiotic biosynthesis by specified mutations in the rpoB gene (encoding the RNA polymerase *β* subunit) of *Streptomyces lividans*. *Journal of Bacteriology*.

[94] Hu H, Ochi K (2001). Novel approach for improving the productivity of antibiotic-producing strains by inducing combined resistant mutations. *Applied and Environmental Microbiology*.

[95] Tamehiro N, Hosaka T, Xu J, Hu H, Otake N, Ochi K (2003). Innovative approach for improvement of an antibiotic-overproducing industrial strain of *Streptomyces albus*. *Applied and Environmental Microbiology*.

[96] Wang G, Hosaka T, Ochi K (2008). Dramatic activation of antibiotic production in *Streptomyces coelicolor* by cumulative drug resistance mutations. *Applied and Environmental Microbiology*.

[97] Xu J, Tozawa Y, Lai C, Hayashi H, Ochi K (2002). A rifampicin resistance mutation in the rpoB gene confers ppGpp-independent antibiotic production in *Streptomyces coelicolor* A3(2). *Molecular Genetics and Genomics*.

[98] Ochi K, Okamoto S, Tozawa Y (2004). Ribosome engineering and secondary metabolite production. *Advances in Applied Microbiology*.

[99] Ochi K (2007). From microbial differentiation to ribosome engineering. *Bioscience, Biotechnology and Biochemistry*.

[100] Hosaka T, Xu J, Ochi K (2006). Increased expression of ribosome recycling factor is responsible for the enhanced protein synthesis during the late growth phase in an antibiotic-overproducing *Streptomyces coelicolor* ribosomal *rpsL* mutant. *Molecular Microbiology*.

[101] Li L, Guo J, Wen Y, Chen Z, Song Y, Li J (2010). Overexpression of ribosome recycling factor causes increased production of avermectin in *Streptomyces avermitilis* strains. *Journal of Industrial Microbiology and Biotechnology*.

[102] Okamoto S, Tamaru A, Nakajima C (2007). Loss of a conserved 7-methylguanosine modification in 16S rRNA confers low-level streptomycin resistance in bacteria. *Molecular Microbiology*.

[103] Nishimura K, Hosaka T, Tokuyama S, Okamoto S, Ochi K (2007). Mutations in rsmG, encoding a 16S rRNA methyltransferase, result in low-level streptomycin resistance and antibiotic overproduction in *Streptomyces coelicolor* A3(2). *Journal of Bacteriology*.

[104] Wang L, Zhao Y, Liu Q, Huang Y, Hu C, Liao G (2012). Improvement of A21978C production in *Streptomyces roseosporus* by reporter-guided rpsL mutation selection. *Journal of Applied Microbiology*.

[105] Huang J, Lih C-J, Pan K-H, Cohen SN (2001). Global analysis of growth phase responsive gene expression and regulation of antibiotic biosynthetic pathways in *Streptomyces coelicolor* using DNA microarrays. *Genes & Development*.

[106] Niraula NP, Kim S-H, Sohng JK, Kim E-S (2010). Biotechnological doxorubicin production: pathway and regulation engineering of strains for enhanced production. *Applied Microbiology and Biotechnology*.

[107] Noh J-H, Kim S-H, Lee H-N, Lee SY, Kim E-S (2010). Isolation and genetic manipulation of the antibiotic down-regulatory gene, wblA ortholog for doxorubicin-producing *Streptomyces* strain improvement. *Applied Microbiology and Biotechnology*.

[108] Chen L, Chen J, Jiang Y, Zhang W, Jiang W, Lu Y (2009). Transcriptomics analyses reveal global roles of the regulator AveI in *Streptomyces avermitilis*. *FEMS Microbiology Letters*.

[109] Duong CTP, Lee H-N, Choi S-S, Lee SY, Kim E-S (2009). Functional expression of SAV3818, a putative TetR-family transcriptional regulatory gene from *Streptomyces avermitilis*, stimulates antibiotic production in *Streptomyces* species. *Journal of Microbiology and Biotechnology*.

[110] Kang S-H, Huang J, Lee H-N, Hur Y-A, Cohen SN, Kim E-S (2007). Interspecies DNA microarray analysis identifies WblA as a pleiotropic down-regulator of antibiotic biosynthesis in *Streptomyces*. *Journal of Bacteriology*.

[111] Kim YJ, Song JY, Moon MH, Smith CP, Hong S-K, Chang YK (2007). pH shock induces overexpression of regulatory and biosynthetic genes for actinorhodin productionin *Streptomyces coelicolor* A3(2). *Applied Microbiology and Biotechnology*.

[112] Lian W, Jayapal KP, Charaniya S (2008). Genome-wide transcriptome analysis reveals that a pleiotropic antibiotic regulator, AfsS, modulates nutritional stress response in *Streptomyces coelicolor* A3(2). *BMC Genomics*.

[113] Lee H-N, Im J-H, Lee M-J, Lee SY, Kim E-S (2009). A putative secreted solute binding protein, SCO6569 is a possible AfsR2-dependent down-regulator of actinorhodin biosynthesis in *Streptomyces coelicolor*. *Process Biochemistry*.

[114] Vohradsky J, Li X-M, Dale G (2000). Developmental control of stress stimulons in *Streptomyces coelicolor* revealed by statistical analyses of global gene expression patterns. *Journal of Bacteriology*.

[115] Hesketh AR, Chandra G, Shaw AD (2002). Primary and secondary metabolism, and post-translational protein modifications, as portrayed by proteomic analysis of *Streptomyces coelicolor*. *Molecular Microbiology*.

[116] Herron PR, Evans MC, Dyson PJ (1999). Low target site specificity of an IS6100-based mini-transposon, Tn1792, developed for transposon mutagenesis of antibiotic-producing *Streptomyces*. *FEMS Microbiology Letters*.

[117] Gehring AM, Nodwell JR, Beverley SM, Losick R (2000). Genomewide insertional mutagenesis in *Streptomyces coelicolor* reveals additional genes involved in morphological differentiation. *Proceedings of the National Academy of Sciences of the United States of America*.

[118] Gust B, Challis GL, Fowler K, Kieser T, Chater KF (2003). PCR-targeted *Streptomyces* gene replacement identifies a protein domain needed for biosynthesis of the sesquiterpene soil odor geosmin. *Proceedings of the National Academy of Sciences of the United States of America*.

[119] Patnaik R (2008). Engineering complex phenotypes in industrial strains. *Biotechnology Progress*.

[120] Askenazi M, Driggers EM, Holtzman DA (2003). Integrating transcriptional and metabolite profiles to direct the engineering of lovastatin-producing fungal strains. *Nature Biotechnology*.

[121] Gao H, Zhou X, Gou Z (2010). Rational design for over-production of desirable microbial metabolites by precision engineering. *Antonie van Leeuwenhoek*.

[122] Tyo KEJ, Ajikumar PK, Stephanopoulos G (2009). Stabilized gene duplication enables long-term selection-free heterologous pathway expression. *Nature Biotechnology*.

[123] Kachroo AH, Jayaram M, Rowley PA (2009). Metabolic engineering without plasmids. *Nature Biotechnology*.

[124] Zhang L, Zhao G, Ding X (2011). Tandem assembly of the epothilone biosynthetic gene cluster by in vitro site-specific recombination. *Scientific Reports*.

[125] Pfeifer BA, Admiraal SJ, Gramajo H, Cane DE, Khosla C (2001). Biosynthesis of complex polyketides in a metabolically engineered strain of *E. coli*. *Science*.

[126] Watanabe K, Hotta K, Praseuth AP (2006). Total biosynthesis of antitumor nonribosomal peptides in *Escherichia coli*. *Nature Chemical Biology*.

[127] Xu P, Vansiri A, Bhan N, Koffas MAG (2012). ePathBrick: a synthetic biology platform for engineering metabolic pathways in E. coli. *ACS Synthetic Biology*.

[128] Chaudhary AK, Park JW, Yoon YJ, Kim BG, Sohng JK (2013). Re-engineering of genetic circuit for 2-deoxystreptamine (2-DOS) biosynthesis in *Escherichia coli* BL21 (DE3). *Biotechnology Letters*.

[129] Li M, Wang J, Geng Y (2012). A strategy of gene overexpression based on tandem repetitive promoters in *Escherichia coli*. *Microbial Cell Factories*.

[130] Baek JM, Mazumdar S, Lee SW Butyrate production in engineered *Escherichia coli* with synthetic scaffolds. *Biotechnology and Bioengineering*.

[131] Conrado RJ, Wu GC, Boock JT (2012). DNA-guided assembly of biosynthetic pathways promotes improved catalytic efficiency. *Nucleic Acids Research*.

[132] Lee JH, Jung SC, Bui LM, Kang KH, Song JJ, Kim SC (2013). Improved production of L-threonine in *Escherichia coli* by use of a DNA scaffold system. *Applied and Environmental Microbiology*.

